# Effect of high-intensity laser therapy and photobiomodulation therapy on oral lichen planus—a systematic review and meta-analysis

**DOI:** 10.1007/s10103-025-04398-8

**Published:** 2025-03-20

**Authors:** Panpan Liu, Qi Zhou, Jie Bao, Muni Chen, Mengting Xu, Jiamin Bian, Yueqiang Wen, Jiayu Yan

**Affiliations:** 1 School of Clinical Medicine, Chengdu University of Traditional Chinese Medicine, Chengdu, China; 2 Sichuan Integrative Medicine Hospital, Chengdu, China; 3 West China Hospital of Stomatology Sichuan University, Chengdu, China; 4 School of Basic Medicine, Chengdu University of Traditional Chinese Medicine, Chengdu, China

**Keywords:** High-intensity laser therapy, Photobiomodulation, Oral lichen planus, Meta-analysis, Systematic review

## Abstract

This study evaluates the efficacy and safety of High-Intensity Laser Therapy (HILT) and Photobiomodulation (PBM) in treating Oral Lichen Planus (OLP), and explores optimal PBM parameter settings. A search of PubMed, Embase, Cochrane Library, Web of Science, CNKI, and SinoMed was conducted for randomized controlled trials (RCTs) on laser therapy for OLP up to October 30, 2024. Study quality was assessed using the Cochrane risk of bias tool and the Jadad score. Eighteen studies were identified, with 16 involving 742 participants included in the meta-analysis. Eleven studies compared PBM with topical corticosteroids (TCS), and five compared HILT with TCS. HILT provided superior pain relief compared to TCS (SMD = -0.51, 95% CI [-0.79,-0.23]), while PBM showed comparable outcomes (SMD = -0.41, 95% CI [-0.87, 0.04]). PBM was more effective at pain relief with total energy density below 120 J/cm^2^. Both HILT and PBM reduced recurrence rates (RR = 0.33,95%CI [0.15,0.73]; RR = 0.43, 95% CI [0.25, 0.74]) and improved cure rates (RR = 1.44, 95% CI [1.01, 2.06]; RR = 1.47, 95% CI [1.05, 2.05]). PBM had no adverse reactions, while HILT and TCS had associated adverse events. HILT and PBM may be considered effective alternatives to TCS. For PBM treatment of OLP, a total energy density below 120 J/cm^2^ is advisable. Further large-scale studies are required to confirm these findings and refine laser parameters.

**Supplementary Information:**

The online version contains supplementary material available at 10.1007/s10103-025-04398-8.

## Introduction

Oral Lichen Planus (OLP), a chronic inflammatory oral mucosal disorder mediated by T cells, presents symmetrical white or reticular striations on the buccal mucosa [1, 2]. It is clinically categorized into reticular, papular, plaque, erosive, atrophic, and bullous types [3]. The erosive and atrophic forms are often associated with pain and a burning sensation, and they may transform into oral squamous cell carcinoma [4]. Consequently, the World Health Organization classifies OLP as a potentially malignant oral condition. The global prevalence of OLP is approximately 0.98% [5], and the malignant transformation rate is 1.14% [6].

At present, corticosteroids and calcineurin inhibitors are the main treatment methods[3]. They have shown significant efficacy in alleviating symptoms such as pain and erosion in OLP patients. However, long-term use may lead to drug resistance and adverse reactions such as xerostomia and oral burning sensation [7, 8]. Additionally, alternative therapies have shown some efficacy in alleviating OLP symptoms. These include natural substances and supplements such as curcumin, chamomile, aloe vera, honey, and zinc acetate[9, 10]. However, the scientific validity of these alternative therapies still needs further investigation[11, 12].

In recent years, HILT and PBM have gained prominence as treatments for various oral diseases such as leukoplakia, periodontitis, and oral cancer. HILT employs photothermal effects of lasers to coagulate, vaporize, and excise diseased tissue, thus influencing cell proliferation and growth factor release, which in turn reduces pain and promotes healing of affected soft tissues in the oral cavity [13]. PBM, also known as low-level laser therapy, enhances wound healing and reduces pain and inflammation by stimulating β-endorphin secretion, increasing epidermal growth factor (EGF) expression, and reducing inflammatory cytokine levels [14]. However, debate continues over whether HILT and PBM can serve as alternatives to TCS for patients with OLP. A meta-analysis by Mahuli et al. [15] indicated that PBM is more effective in treating OLP, while Soh et al. [16] contended that PBM does not provide a distinct advantage over TCS. Moreover, the study by Soh et al. involved only 317 patients and included observational studies, which limits the reliability of their findings. Meanwhile, Mahuli et al. performed a quantitative analysis on only three outcomes without conducting subgroup analyses based on the number of laser treatment sessions or patient types. This limitation restricts the applicability of the evidence. Regarding HILT studies [17], only a systematic review is available, with no meta-analysis conducted to date.

Given the limitations of prior meta-analyses and the increase in published RCTs [18–22], this study undertakes a comprehensive systematic review and meta-analysis to evaluate and compare the effectiveness and safety of HILT and TCS for managing OLP, and to assess the relative effectiveness of PBM compared to TCS. Additionally, this review aims to identify the most effective intervention parameters for PBM, thereby providing clinicians with a broader range of therapeutic options.

## Methods

### Protocol

The study protocol adhered to the PRISMA guidelines (Table S1) [23]. The protocol was registered with PROSPERO (Registration No. CRD42024531390), and discrepancies between the initial registered protocol and the published manuscript are elaborated in Supplementary File 1.

### Search strategy

A comprehensive search was conducted across PubMed, Web of Science, Cochrane Library, Embase, CNKI, and SinoMed to retrieve articles on HILT and PBM therapy for OLP from the inception of each database until October 30, 2024. No language restrictions were imposed. Search terms included “OLP,” “lichen planus,” “oral,” “HILT,” “Low-Level Light Therapy,” “photobiomodulation Therapy,” and “laser therapy.” Specific modalities such as CO2 Laser and Nd:YAG laser were also included. Boolean operators “OR” were used to link different interventions, and “AND” to connect diseases with interventions. A manual search of references and systematic reviews was also conducted to ensure comprehensiveness. The search strategy is detailed in Supplementary File 1.

### Inclusion and exclusion criteria

Inclusion criteria were established using the PICOS framework: P (patients) were those with OLP confirmed by clinical or histopathological diagnosis; I (intervention) involved treatments with PBM or HILT; C (comparator) comprised OLP patients receiving non-laser therapies; O (outcomes) included measurements of pain, clinical scores, recurrence rate, cure rate, and adverse reactions; S (study) specified that only RCT were included. Exclusion criteria included non-RCTs, animal studies, in vitro studies, case reports, letters, and unpublished manuscripts.

Pain levels were quantified using the Visual Analog Scale (VAS), ranging from 0 (no pain) to 10 (maximum pain). Clinical outcomes were assessed using the Thongprasom sign score (TSS) for single lesions [24], or the Reticular-Atrophic-Erosive/ulcerative (RAE) score for multiple lesions [25]. Cure rates were evaluated through efficacy indices (EI), which identified patients experiencing complete healing or resolution of pain and lesions. Reported adverse events included oral sensory disturbances (such as dysgeusia, burning sensation, piercing pain, dry mouth), fungal infections (such as Candida infection), gastrointestinal discomfort (such as nausea, gastrointestinal upset), and other noted side effects.

### Literature screening

Initially, P.P.L. and Q.Z. independently imported the retrieved studies into EndNote 20 and removed any duplicates. They then conducted a preliminary screening by reviewing titles and abstracts to eliminate non-clinical studies that did not relate to HILT and PBM for OLP. The full texts of the remaining studies were further evaluated by both researchers to determine eligibility for inclusion. In cases of disagreement, the corresponding author (J.Y.Y.) mediated to achieve consensus.

### Quality assessments and data extraction

Two authors conducted an independent assessment of the risk of bias for all included RCTs using the Cochrane Risk of Bias tool [26]. The evaluation covered seven domains: random sequence generation, allocation concealment, blinding of participants and personnel, blinding of outcome assessment, completeness of outcome data, selective reporting, and other potential biases. Each domain was assigned a risk level of high, unclear, or low. Additionally, the Jadad score [27], which rates studies on randomization, allocation concealment, blinding, and the handling of withdrawals and dropouts, was employed to further assess study quality. Studies scoring between 1 and 3 were deemed low quality, whereas scores from 4 to 7 indicated high quality. Plots summarizing the risk of bias were generated using Review Manager (Version 5.4) [28].

Data including the first author, publication year, study country, OLP type, sample size, participant sex and age, interventions, outcomes, and study duration were extracted independently by L.P.P. and Z.Q. Treatment details such as laser type, wavelength, mode and duration of irradiation, power density, output power, energy fluence, and treatment frequency were also recorded. Any discrepancies were resolved by consulting a third reviewer (J.Y.Y.).

### Statistical analysis

Data analysis was conducted using STATA 15 [29]. Dichotomous outcomes were analyzed using relative risk (RR), while continuous outcomes were assessed with standardized mean difference (SMD) and 95% confidence interval (CI). The I^2^ index assessed heterogeneity; an I^2^ ≤ 50% with *P* > 0.05 suggested low heterogeneity and warranted a fixed-effects model. Conversely, an I^2^ > 50% with *P* ≤ 0.05 indicated significant heterogeneity, necessitating a random-effects model [30]. Data transformations were applied as per Luo et al. [31] for converting non-normally distributed values into normally distributed data. McGrath et al.’s [32] formula was utilized for converting other non-normally distributed data. Missing standard deviations were estimated using values from other groups in the same study as per Furukawa et al. [33]. Studies lacking both mean and standard deviation were excluded. Sensitivity analysis tested the robustness of the finding.

### Subgroup analyses

Subgroup analyses were conducted to compare therapeutic effects between patients undergoing PBM and those receiving TCS, categorized by patient type, laser wavelength, intervention frequency, control group medication type,Number of lesions and follow-up time.

### Publication bias

Publication bias was assessed using Egger’s test when five or more studies were available[34].

## Results

### Search results

**Fig. 1:**
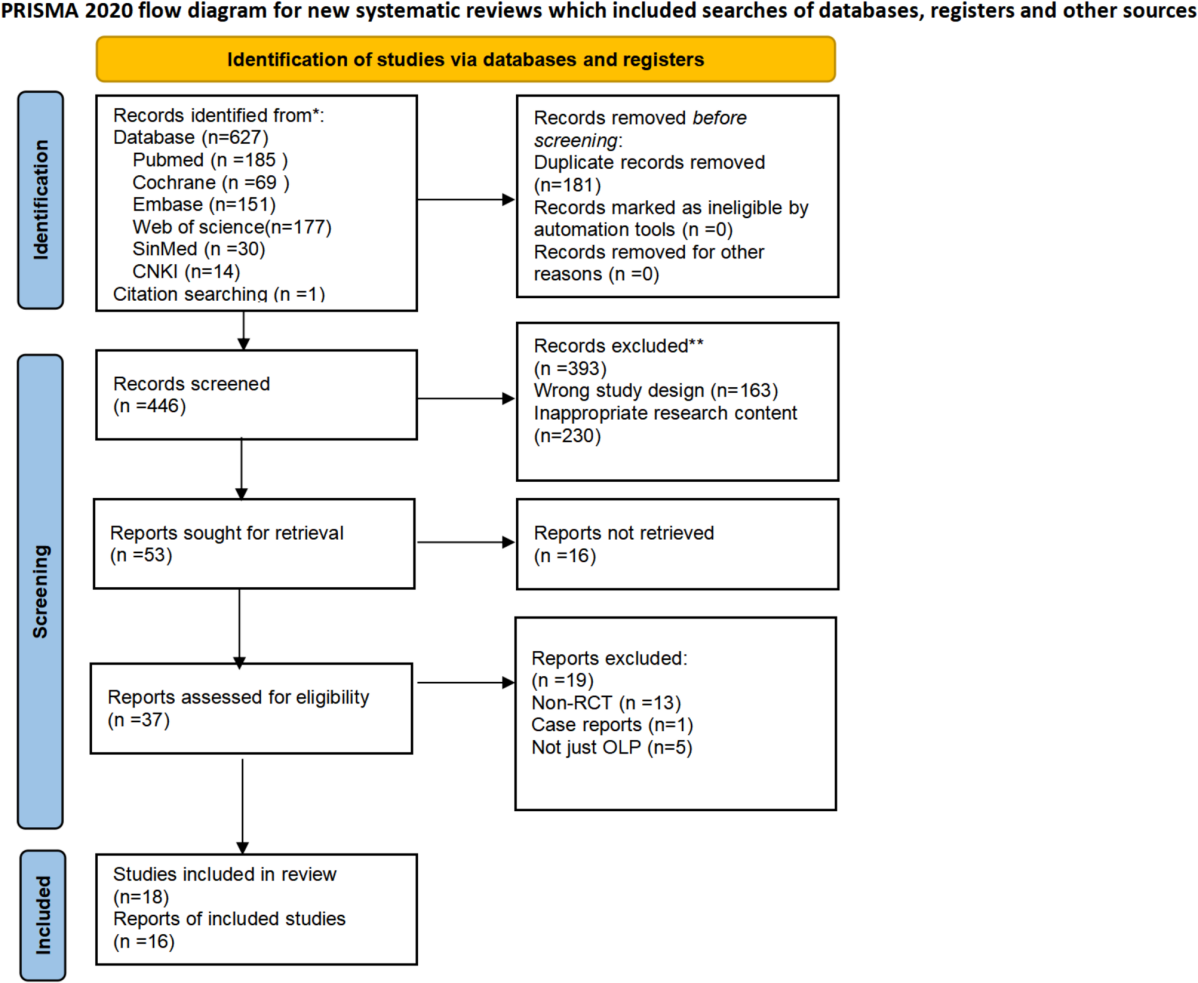
PRISMA flow diagram

Using the specified search strategy, a total of 627 articles were retrieved. Of these, 626 articles were sourced from six databases, and one article was identified through citation tracking. After removing 181 duplicate articles, titles and abstracts of the remaining articles were screened, leading to the exclusion of review articles, case reports, studies with unrelated content, and non-RCTs, eliminating 393 articles. After this initial screening, 53 articles remained. Upon further review of the full texts based on the inclusion criteria, 35 articles were excluded. Among these, 16 articles were registered studies with no full text available, 13 were non-RCTs, 5 addressed not only OLP but also oral leukoplakia and other oral potentially malignant disorders, and 1 was a case report. Ultimately, 18 were eligible for systematic review, with 16 included in the meta-analysis. Figure 1 illustrates the search process and selection criteria.

### Characteristics of included studies

Since Sanjay et al.’s research [35] included two experimental groups, it was divided into Sanjay et al. 2022a and Sanjay et al. 2022b, with the control group divided accordingly. In total, 18 studies [18–22, 25, 35–46] were included, involving 742 patients from ten countries and regions. Four studies originated from China [43–46], three from India [19, 22, 35], two from Brazil [40, 42], Iran [21, 38], and Egypt [20, 39], and one each from Syria [18], Russia, Pakistan [25], Turkey [37], and Spain [41]. The number of participants ranged from 8 to 120, with follow-up durations varying from one month to two years. Among all studies in the meta-analysis, five studies combined laser therapy with TCS in the experimental group [18, 35, 43–45], and one study used two different TCS in the control group [35]. The remaining studies utilized a single laser therapy in the experimental group and a TCS in the control group. Our TCS included 0.1% triamcinolone, 0.5 mg dexamethasone, 0.05% clobetasol propionate, and 0.5 mg betamethasone. Additionally, to prevent oral candidiasis induced by TCS, three studies in the control group used nystatin solution [37, 38, 40], and one study used miconazole oral gel [20]. Table 1 presents all key characteristics of the eligible research.

**Table 1:** Baseline characteristics of the included studies

Study	Country	Participants	Study design	Study groups	Sample description (male/female); age (mean ± SD or median (minimum — maximum)];	Experimental group	Control group	Outcome	Jadad scale
Mohamed et al. 2024 [20]	Egypt	Erosive OLP	RCT	PBM(*n* = 22) TCS(= 22)	52.91 ± 12.41; NA	980 nm diode laser, BiW,for 5 weeks	0.1% topical TA Tid plus miconazole oral gel qd,4 wks	Pain,Clinical scores,	5
Sanjay et al. 2022 [35]	India	Symptomatic OLP	3-arm-RCT	PBM(*n* = 10) TCS(= 10) PBM plus TCS(= 10)	NA; 18–60	PBM with 904 nm GaAs laser PBM and TCS, All groups for 15 days in 5 sessions	0.5mg BMV/10ml waster, Gargle for 1 week, followed by TA oral paste (0.1%)	Pain (VAS) and healing WHO oral mucositis assessment scale,	3
Mirza et al. 2018 [25]	Pakistan	Erosive-Atrophic OLP	3-arm-RCT	PBM(*n* = 15) TCS(= 15)	8/37 PBM:50.8 ± 14.7 TCS:49.2 ± 10.6	diode laser 630 nm, 10 mW, BiW for 1 months	(1) Dex 5ml water, 5ml water, qid × 1month	Sign score changes, pain improvement, efficacy index	5
Wang et al.2017 [44]	china	Symptomatic OLP	RCT	TCS plus PBM(*n* = 40) TCS (*n* = 40)	34/46 TCS plus PBM:42.62 ± 2.3 TCS:39.48 ± 1.7	dioder laser three weekly plus TA ointment,1x/day for 4 wks	TA ointment,1x/day for 4 wks	VAS, clinical resolution	2
Kazancioglu et al. 2015 [37]	Turkey	Erosive-Atrophic OLP	4-arm-RCT	PBM(*n* = 30) TCS(*n* = 30)	NA; 42.6 ± 8.3	Diode laser:2.5 min/time,BIW, maximum10 sessions	Dex mouth wash for 5 min, then Nystatin rinse (30 drops, 100,000 units) after 30 min, qid × 1 month	Response rate scores appearance and pain	5
Jajarm et al. 2011 [38]	Iran	Erosive-Atrophic OLP	RCT	PBM(*n* = 15) TCS(= 15)	NA;NA	dioder laser 2.5 min/time,two times a week	Dex 0.5mg/5ml qid × 5min, followed by Nystatin 30 drops qid × 5min, for 1 month	Appearance score, pain score, lesion severity	3
Shenawy et al. 2018 [39]	Egypt	Erosive-Atrophic OLP	RCT	PBM(*n* = 12) TCS(= 12)	5/19; PBM:52.2 ± 6.4 TCS:53.6 ± 13.2	dioder laser tiw for 2 months, for a maximum of 10 sessions	0.1% TA gel qid, 4wks; if extension, Miconazole gel qid × 1wk	Pain (VAS), RAE	4
Dillenburg et al. 2014 [40]	Brazil	Erosive-Atrophic OLP	RCT	PBM(*n* = 21) TCS(= 21)	7/35; 58.2 ± 14.23	InGaAlP diode laser irradiation three times a week for four weeks	Clobetasol Propionate gel 0.05%bid × 30 days nystatin	Clinical scores, symptom scores Recurrence,pain (VAS)	3
Panchal et al. 2023 [22]	India	erosive OLP	RCT	PBM plus TCS (*n* = 30) TCS (*n* = 30)	20/40; 18–60	PBM: 10 min, twice weekly for 9 sessions	0.1% TA ointment applied 5 times daily for 28 days	Pain, clinical remission,	2
Salinas-Gilabert et al. 2023 [41]	Spain	Symptomatic OLP	Arm-RCT	PBM(*n* = 20) TCS(= 19)	NA;60.7 ± 9.7	Low-power laser once a week for 4 sessions	(1)TCS: TA cream 0.1% tid × 1 month + inactive laser once a week for 4 sessions	Pain, Thongprasom severity score,	7
Ferri et al. 2021 [42]	Brazil	Symptomatic OLP	RCT	PBM(*n* = 17) TCS(= 17)	2/32; 30–83	GaAIAs diode laser, twice weekly 8 sessions, gel placebo × 30 days	The CP gel 0.05%,tid × 1month plus tiw × 1months of laser placebo	Pain, clinical scores, clinical rate	7
Wang et al.2022 [43]	china	Erosive OLP	3-arm-RCT	A: TCS 1(*n* = 29) B:TCS 2(*n* = 30) C:HILT plus TCS(*n* = 28)	21/66 A:47.27 ± 6.93 B:49.17 ± 11.86 C:50.47 ± 11.88	ND:YAG,twice weekly for 1 month	A:topical TAOintment B:Compound clobetasol propionate Ointment bid × 4wks	TSS, VAS,Adverse reaction, recurrence	3
Zhong et al.2020 [45]	china	Erosive OLP	RCT	HILT plus TCS(*n* = 32) TCS (*n* = 32)	34/30 HILT plus TCS:42.38 ± 3.18 TCS:45.87 ± 5.42	ND:YAG once every 3 weeks,no more than 3 times Plus TA unguent Twice a day	TA ointment bid	effective rate adverse reactions and recurrence	3
Liu et al.2023 [46]	china	Erosive OLP	RCT	HILT (*n* = 20) TCS (*n* = 20)	9/31 HILT:58.6 ± 10.4 TCS:59.9 ± 12.1	Er:YAG,once weekly (no more than 10 sessions)	TA ointment Tid,for a maximum of 30 days	TSS,VAS,recurrence,Effective rate	2
Ibrahim et al. 2023 [18]	Syria	Symptomatic OLP	Split-mouth RCT	HILT (*n* = 16) TCS (*n* = 16)	6/10;44.8 ± 12.6	CO2 laser vaporisation, 1 session	TA intralesional injection, 40mg/mL, once a week for 4 wks	TSS, VAS, lesion area	3
Bhatt et al. 2022 [19]	India	OLP	RCT	PBM(*n* = 30) Antioxidant(= 30)	22/38; PBM:42.47 ± 13.01 Antioxidant:39.00 ± 15.11	PBM, 980 nm, twice weekly for 2 months	Topical aloe vera gel, applied thrice daily for 2 months	VAS, site score, severity score	4
Tarasenko et al. 2021 [36]	Russia	Erosive OLP	4-arm-RCT	HILT1 (*n* = 19) HILT2 (*n* = 15) HILT3 (*n* = 20) Scapel(*n* = 21)	NA;NA	(1) Er:YAG, (2)Nd:YAG,(3)Er:YAG combination Nd:YAG for ablation and coagulation 1 session at begining	Scalpel excision	pain level, time of epithelization	4
Khalighi et al. 2022 [21]	Iran	Symptomatic OLP	Split-mouth RCT	HILT (*n* = 8) TCS (*n* = 8)	NA;NA	Er,Cr:YSGGlaser,Once a week for 8 sessions	Only TA ointment for 8 weeks	VAS;Thongprasom scale	6

Abbreviations: *OLP* Oral lichen planus, *RCT* Randomized controlled trial, *PBM* Photobiomodulation, *HILT* Fhigh-level laser therapy, *TCS* Topical corticosteroids, *BID* Tow times a day, *QID* Four times a day, *TID* Three times daily, *QD* Once daily, *BiW* Twice weekly, *NA* Not available, *NR* Not reported, *VAS* Visual analogue scale, *RAE* Score:reticular-atrophic-erosive score, *REU* Score:reticular–erythematous–ulcerative score, *PDT* Antimicrobial photodynamic therapy, *BMV* Betamethasone Valerate, *Dex* Dexamethasone, *WKS* Weeks, *CP* Clobetasol propionate, *GaAlAs* Gallium-Aluminum-Arsenide, *TA* Triamcinolone acetonide, *TSS* Thongprasom sign scoring

### Laser-associated outcomes

Among the included studies, six employed HILT, with one being a 4-arm RCT. Twelve studies used PBM, with one being a three-arm trial. The parameters for laser use varied widely. The wavelength range for HILT was 1,064 to 10,600 nm, while for PBM, it was 630 to 980 nm. The features of lasers are displayed in Table 2.

**Table 2:** Laser signature table

Study	Laser type	Wavelength (nm)	Irradiation mode	Irradiation time	Power density (mW/cm^2^)	Power output (w)	Energy fluence (J/cm^2^)	Sessions of laser application
Mohamed et al. 2024 [20]	diode laser	980	non-contact	4 s per point	NR	0.3	1.2	10
Sanjay et al. 2022 [35]	GaAs	904	direct contact	2 min	NR	NR	NR	5
Mirza et al. 2018 [25]	diode laser	630	continuous wave	2.5 min	10	0.01	1.5 per session	10
Wang et al.2017 [44]	diode laser	810	non-contact	2.25 min	1.56	0.6	NR	12
Kazancioglu et al. 2015 [37]	Diode laser	808	continuous wave	2.5 min	10	0.1	120	10
Jajarm et al. 2011 [38]	Diode laser	630	continuous wave	2.5 min	10	0.01	1.5	10
Shenawy et al. 2018 [39]	diode laser	970	continuous wave non-contact	2 min	NR	2	180 J (total)	10
Dillenburg et al. 2014 [40]	InGaAlP diode laser	660	continuous wave non-contact	6 s per point	1000	0.04	6	12
Panchal et al. 2023 [22]	diode laser	810	continuous wave non-contact	10 min	NR	0.8–0.9	NR	9
Salinas-Gilabert et al. 2023 [41]	PBM	NR	NR	30 s per spot	200 (per spot)	NR	6	4
Ferri et al. 2021 [42]	GaAIAs diode laser	660	continuous wave	5 s per point	35.4	0.1	177	8
Wang et al.2022 [43]	Nd:YAG	1064	non-contact	5min	NR	NR	NR	10
Zhong et al.2020 [45]	Nd:YAG	1064		NR	NR	NR	NR	3
Liu et al.2023 [46]	Er: YAG	2940	non-contact	NR	NR	NR	0.05	10
Ibrahim et al. 2023 [18]	CO2 laser	10,600	continuous wave	NR	1,527,800 (total)	3	NR	1
Khalighi et al. 2022 [21]	Er,Cr:YSGG	2780	non-contact	1.5 min	NR	NR	1.75	8
Tarasenko et al. 2021a [36]	Er:YAG	2940	direct contact	NR	NR	2 (ab),3 (co)	NR	1
Tarasenko et al. 2021b [36]	Nd:YAG	1064	non-contact	NR	NR	1.5(ab), 3(co)	NR	1
Tarasenko et al. 2021c [36]	Er:YAG plus Nd:YAG	2940 + 1064	non-contact plus direct contact	NR	NR	2(ab),3(co) plus 1.5 (ab),3(co)	NR	1
Bhatt et al. 2022 [19]	diode laser	980	non-contact	0.33 min	600	0.3	12	16

Abbreviations: *NR* Not reported, *ab* ablation, *co* coagulation

### Risk of bias assessments and quality evaluation

The risk of bias is illustrated in Fig. S1 Specifically, three RCTs exhibited a high risk: one study used a single-blind design [19]; one lacked an adequate description of allocation concealment [18]; and one [22] used a high-risk randomized method. Two RCTs met all criteria and were classified as having a low risk. Thirteen RCTs had an unclear risk due to vague descriptions of one or more items. The study quality was assessed using the Jadad scale. Three RCTs [22, 44, 46] were rated 2 points, six RCTs [18, 35, 38, 40, 43, 45] were rated 3 points, three RCTs [19, 22] were rated 4 points, three RCTs [20, 25, 40] were rated 5 points, one RCT [21] was rated 6 points, and two RCTs [41, 42] were rated 7 points (Jadad scale results are shown in Table 1 of the main characteristics).

### Pain measurement based on VAS scores

**Fig. 2:**
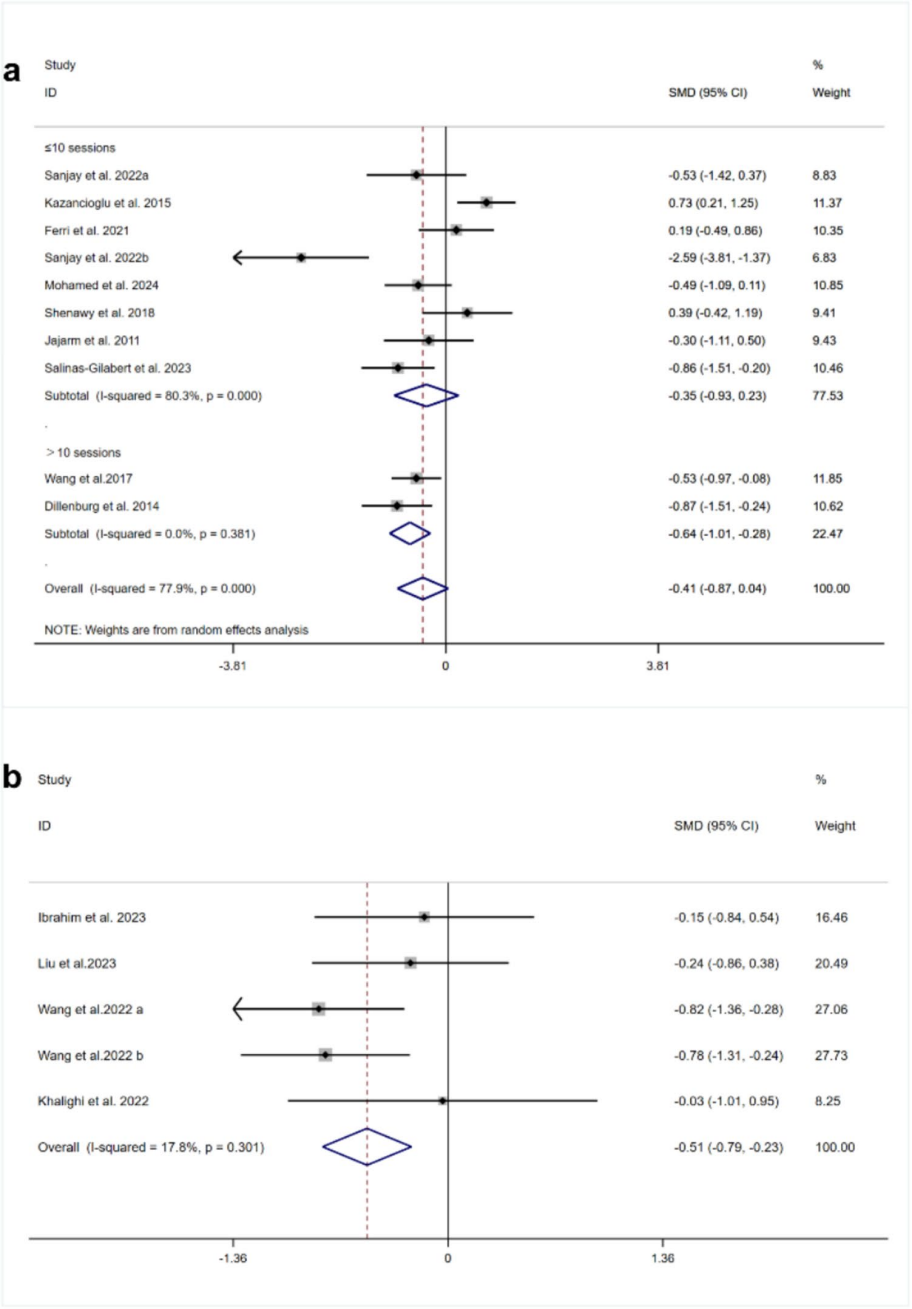
Forest plots of VAS score comparing **a** PBM and TCS **b** HILT and TCS

Thirteen RCTs provided analyzable VAS score data [18, 20, 21, 35, 37–44, 46]. Among these, nine studies reported the VAS scores for PBM treatment of OLP. The overall heterogeneity was high (I^2^ = 77.9%, *p* = 0.000), so a random-effects model was used. The findings revealed no statistically significant difference in the VAS scores between PBM and TCS (SMD = −0.41,95% CI [−0.87, 0.04],  *p*= 0.076) (Fig. 2a).

Four RCTs (*n* = 203) investigated the VAS score following HILT treatment for OLP [18, 21, 43, 46]. Our meta-analysis demonstrated that the VAS scores were notably lower in the HILT group than in the TCS group (SMD = −0.51, 95% CI [−0.79, −0.23], *p* = 0.002, I^2^ = 17.8%) (Fig. 2b).

### Clinical scores

**Fig. 3:**
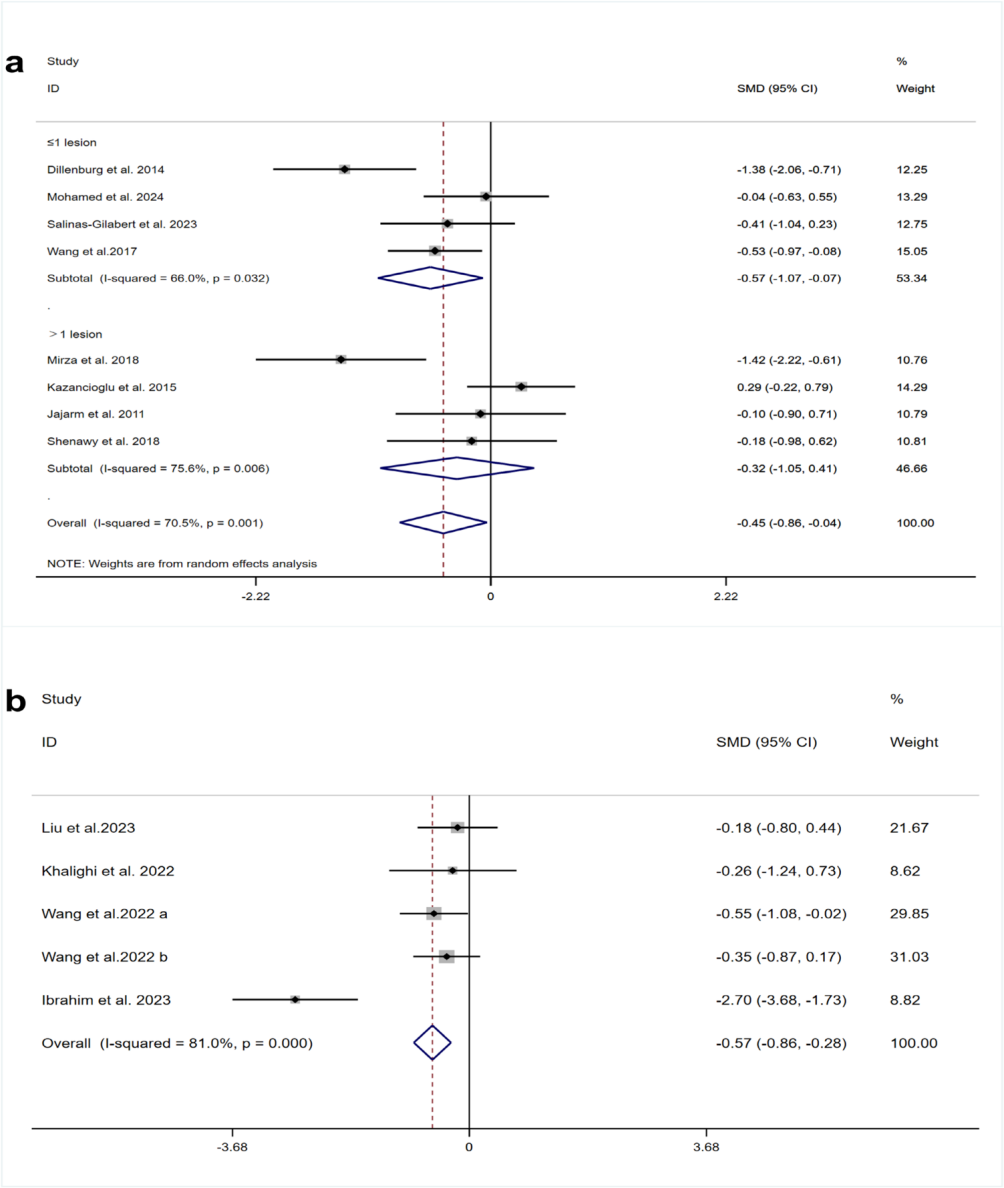
Forest plots of clinical score comparing **a** PBM and TCS **b** HILT and TCS

Eight studies[20, 25, 35, 38, 40, 41, 44] reported clinical scores for PBM treatment. Due to substantial heterogeneity (I^2^ = 70.5%, *p* = 0.001), a random-effects model was applied. Our meta-analysis showed that clinical scores were notably lower in the PBM group than in the steroid group (SMD = −0.45, 95% CI [−0.86, −0.04], *p* = 0.033) (Fig. 3a). Furthermore, subgroup analysis of clinical scores indicated that, with one or fewer lesions, the laser therapy group had lower clinical scores than the TCS group (SMD = −0.57, 95% CI [−1.07, −0.07], *p* = 0.024). In contrast, when more than one lesion was present, there was no statistically significant difference between the groups (SMD = −0.32, 95% CI [−1.05, 0.41], *p* = 0.0388). Four studies [18, 43, 45, 46] on HILT reported clinical outcome scores. The heterogeneity of the included studies was high (I^2^ = 81%, *p *= 0.000), and a random-effects model was applied. The meta-analysis demonstrated that the HILT group had significantly lower clinical scores compared to the TCS group (SMD = −0.57, 95% CI [−0.86, −0.28], *p* = 0.036) (Fig. 3b).

### Cure rate

**Fig. 4:**
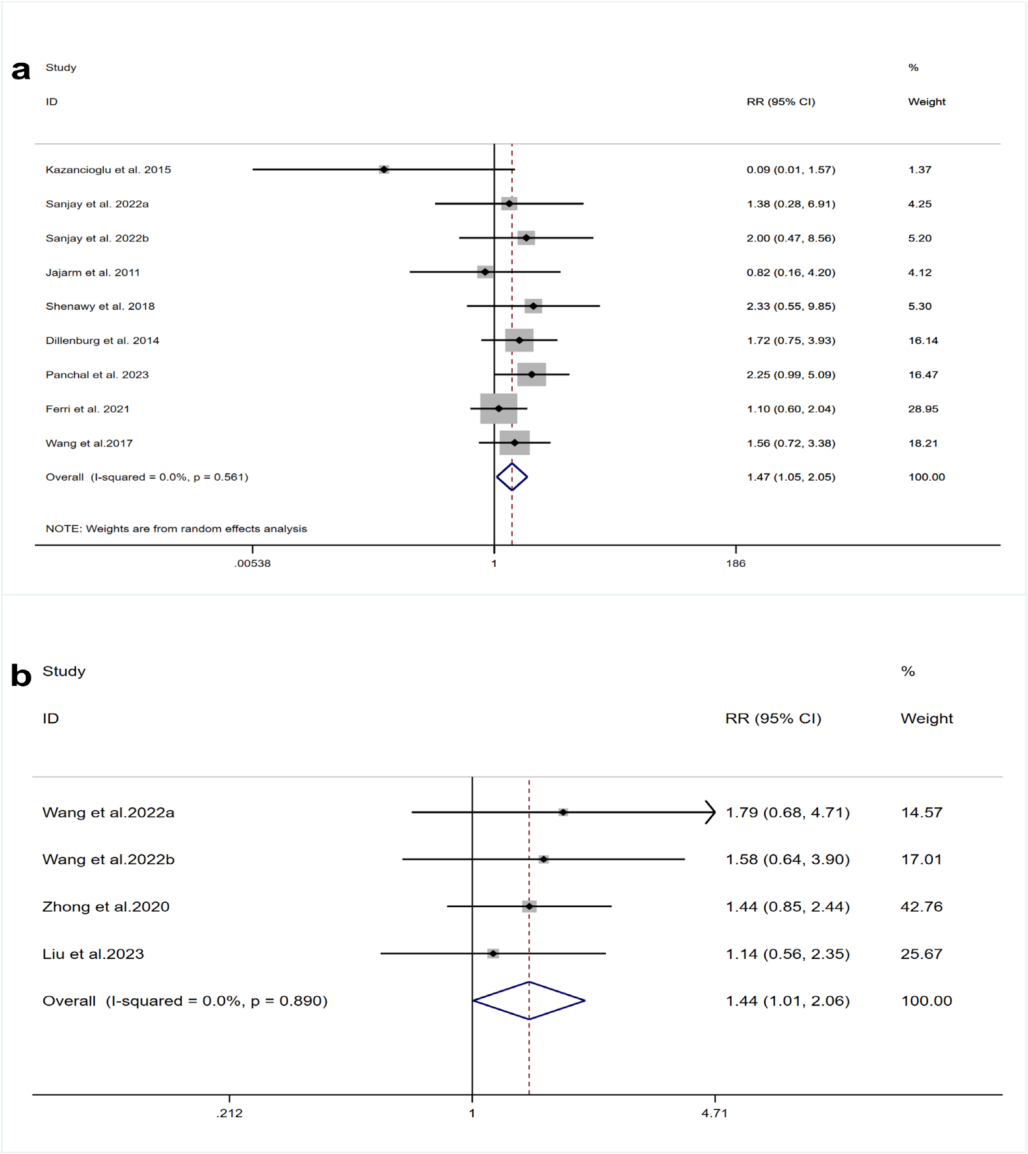
Forest plots of cure rate comparing **a** PBM and TCS **b** HILT and TCS

Data from seven RCTs [22, 35, 37–40, 42] were included to assess cure rates, demonstrating minimal heterogeneity (I^2^ = 0%, *p *= 0.561). The meta-analytical outcomes showed that the PBM group achieved a statistically higher cure rate than the steroid group (RR = 1.47, 95% CI [1.05, 2.05], *p* = 0.023) (Fig. 4a).

Additionally, three RCTs [43, 45, 46] evaluated cure rates using HILT. Analysis indicated negligible heterogeneity among these studies (I^2^ = 0%, *p* = 0.890). Results of the meta-analysis revealed that the HILT group had a significantly elevated rate of treatment success in comparison to the steroid group (RR = 1.44, 95% CI [1.01, 2.06], *p* = 0.047) (Fig. 4b).

### Recurrence rate

**Fig. 5:**
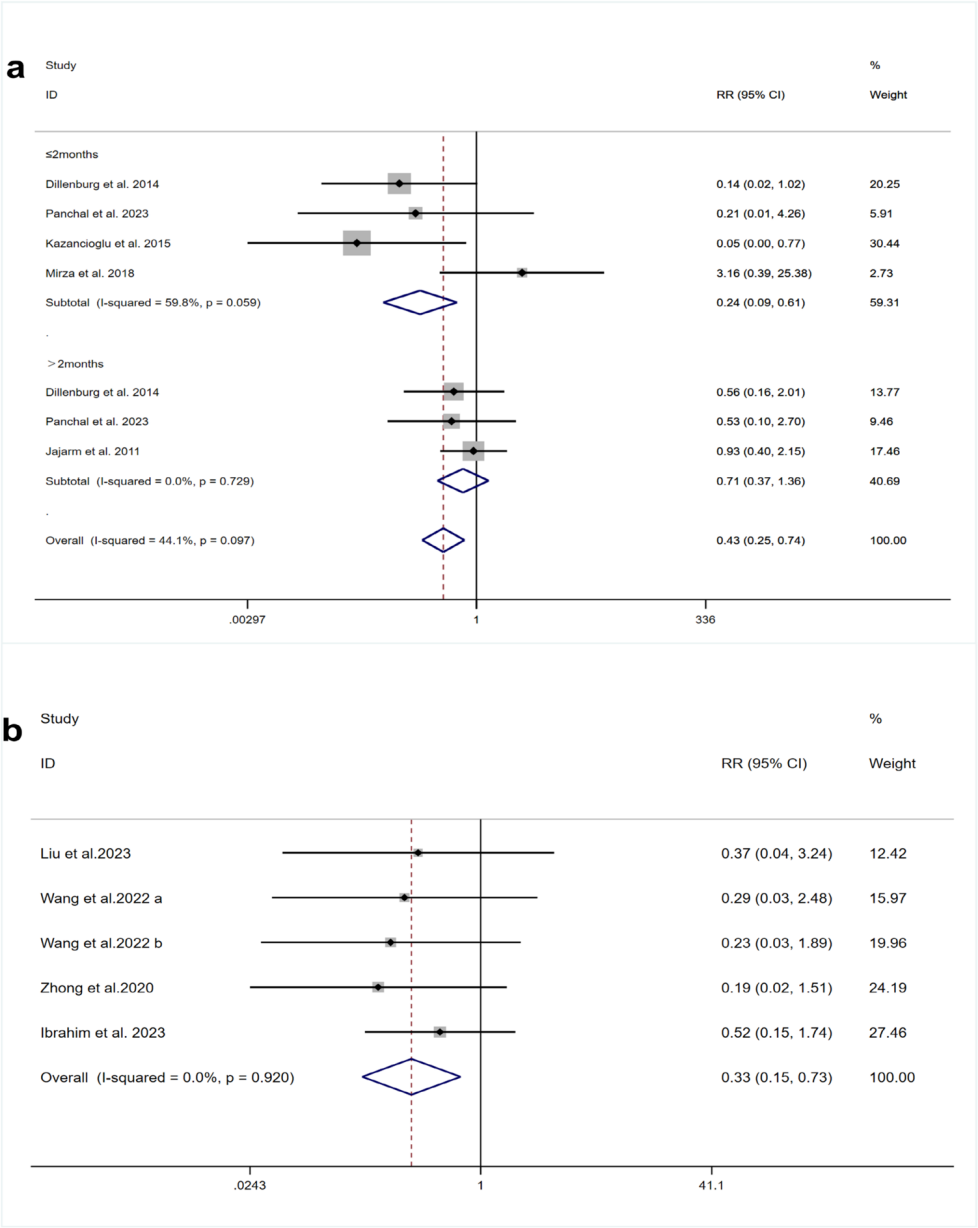
Forest plots of recurrence rate comparing **a** PBM and TCS **b** HILT and TCS

Five RCTs [22, 25, 37, 38, 40] provided data for analyzing recurrence rates in OLP treated with PBM. Moderate heterogeneity was observed among these RCTs (I^2^ = 44.1%, *p* = 0.097). The meta-analysis demonstrated that the PBM treatment resulted in a significantly reduced recurrence rate compared to the TCS group (RR = 0.43, 95%CI [0.25, 0.74], *p* < 0.01). Subgroup analysis for follow-up durations of ≤ 2 months showed a lower recurrence rate in the PBM group relative to the TCS group (RR = 0.24, 95%CI [0.09, 0.61], *p* = 0.003). For follow-up periods longer than 2 months, the difference in recurrence rates was not statistically significant (RR = 0.71, 95%CI [0.37, 1.36], *p* = 0.305) (Fig. 5a).

Four RCTs [18, 43, 45, 46] were analyzed for recurrence rates in OLP treated with HILT. Low heterogeneity was reported among these studies (I^2^ = 0.0%, *p* = 0.920). Meta-analysis indicated that the recurrence rate in the HILT group was significantly lower than in the TCS group (RR = 0.33, 95%CI [0.15, 0.73], *p* = 0.006) (Fig. 5b).

### Adverse events

Adverse reactions related to HILT were documented in two clinical trials [43, 45], showing negligible heterogeneity (I^2^ = 0.0%). A significant reduction in adverse events was noted in the experimental group (*n* = 88) compared with the control group (*n* = 91) (RR = 0.27, 95%CI [0.12, 0.63], *p* = 0.002) (Fig. S2). In contrast, the PBM cohort reported no adverse reactions. A singular study noted that three control group participants initially experienced transient oral burning sensations, and two reported gastrointestinal discomfort [40]. Table S2 enumerates the adverse reactions identified in each study.

### Subgroup analysis

Subgroup analyses evaluated the analgesic efficacy of PBM versus TCS in managing OLP (Table S3). Results indicated that PBM significantly lowered VAS scores in symptomatic OLP patients (SMD = −0.73, 95%CI [−1.38, −0.09], *p* = 0.026). In erosive-atrophic OLP cases, no significant efficacy difference was observed between PBM and TCS treatments (SMD = −0.01, 95%CI [−0.79, 0.78], *p* = 0.985) (Fig. S3). Analysis by the number of treatment sessions revealed no significant VAS score differences with fewer than 10 PBM sessions (SMD = −0.35, 95% CI [−0.93, 0.23], *p* = 0.240). Conversely, more than 10 sessions led to notably lower VAS scores in the PBM group compared to TCS (SMD = −0.64, 95% CI [−1.01, −0.28], *p* = 0.001)**.** No significant differences emerged concerning laser wavelength or medication types in the control group (SMD = −0.36, 95% CI [−0.86, 0.13], *p* = 0.149; SMD = −0.41, 95% CI [−0.87, 0.04], *p* = 0.076) (Figs. S4 and S5).

### Heterogeneity

Significant heterogeneity (I^2^ > 50%) was noted in studies evaluating VAS scores, leading to a subgroup analysis of studies with high heterogeneity. Analyzed factors included laser wavelength (I^2^ = 78.9%; *p* = 0.149), number of laser sessions (I^2^ = 77.9%; *p* = 0.076), OLP types (I^2^ = 80.2%; *p* = 0.118), and medication types in the control group (I^2^ = 77.9%; *p* = 0.076). This analysis indicated that the number of sessions could contribute significantly to heterogeneity. Specifically, greater heterogeneity was detected when the number of sessions was fewer than ten (I^2^ = 80.3%; *p* = 0.240), while it was resolved when sessions exceeded ten (I^2^ = 0.0%; *p* = 0.001), correlating with enhanced therapeutic outcomes. These results imply that session frequency is a crucial factor in heterogeneity.

Moreover, of the nine studies assessing pain relief, only three showed superior outcomes in the TCS group, which were the three sole studies reporting total energy flux values (120 J/cm^2^, 177 J/cm^2^, 180 J/cm^2^). Excluding variables such as wavelength and treatment duration, it was deduced that PBM’s analgesic effect on OLP declines when energy flux exceeds 120 J/cm^2^, suggesting another potential source of heterogeneity. It is essential to acknowledge that the limited sample size might introduce bias in the I^2^ calculation[47].

### Publication bias and sensitivity analysis

Publication bias was evaluated for metrics from studies totaling more than five. Egger’s test suggested an absence of publication bias. Comparisons of PBM and TCS for VAS scores, clinical scores, cure rates, and recurrence rates yielded *p*-values of 0.290, 0.442, 0.941, and 0.922, respectively (Fig. S6). A sensitivity analysis confirmed the robustness of the results across all evaluated outcomes (Figs. S7 and S8).

## Discussion

This study represents the first meta-analysis to evaluate the effectiveness and safety of various HILT treatments for OLP, and compares the efficacy and safety of PBM versus TCS in managing OLP.

Our results indicate that HILT is superior to TCS in reducing pain, lesion severity, and improving cure rates over short-term treatment durations (4–8 weeks). Additionally, HILT demonstrated a lower recurrence rate after more than three months of follow-up compared to TCS. In the same short-term period, PBM matched TCS in improving pain scores and was more effective in clinical severity, recurrence rates, and cure rates. Although no overall significant difference in pain score improvement was noted between PBM and TCS, a subgroup analysis by intervention frequency showed that more than 10 PBM sessions significantly enhanced symptom relief. Conversely, fewer than 10 sessions yielded comparable pain reduction to TCS. Subgroup analysis by patient type indicated that PBM notably reduced VAS scores in symptomatic OLP patients but showed no difference in erosive-atrophic OLP patients. Further analysis based on follow-up duration found PBM more effective in reducing recurrence rates within two months, with no significant differences observed for longer follow-ups. These findings suggest that the efficacy of PBM may depend on intervention frequency, patient type, and treatment duration. Regarding side effects, both HILT and TCS were associated with local and systemic adverse reactions, including oral burning and candidiasis, yet no adverse effects were reported in the PBM group**.**

Previous research on HILT for treating OLP has largely focused on CO2 laser treatments, involving study designs such as cohort studies and case reports, which restrict the generalizability of the findings [17]. This study exclusively incorporated high-quality RCTs and, through a meta-analysis, preliminarily assessed the efficacy and safety of HILT for OLP. Unlike other systematic reviews on PBM treatment for OLP, our review included more recent studies and conducted multiple subgroup analyses on the primary outcome (VAS score). Regarding VAS score improvements, our results align with those of Soh et al. (*n* = 6), confirming that PBM and TCS have comparable efficacy in reducing VAS scores. However, additional subgroup analyses indicated that PBM provided greater pain relief than TCS when the number of laser interventions exceeded 10. A further subgroup analysis on wavelength showed no significant differences in pain score improvements between PBM and TCS. In contrast, Mahuli et al. (*n* = 8) reported that PBM was more effective than TCS at wavelengths above 800 nm, a discrepancy possibly due to the use of a fixed-effect model under high heterogeneity (I^2^ = 68%), which may have underestimated the variations between studies. In terms of clinical scores, we used SMD to TSS and RAE scores, finding that PBM surpassed TCS in clinical outcomes when fewer lesions were present. Both Soh et al. (*n* = 4) and Mahuli et al. (*n* = 5) focused solely on TSS scores and found no significant differences between the interventions, potentially due to the limited number of studies, which restricted the statistical power of the effect size. Data on recurrence and cure rates were scarce, and Soh did not report these outcomes. However, Mahuli et al. noted that PBM and TCS had similar recurrence rates at follow-up periods of 60 to 90 days, consistent with our findings. Moreover, our subgroup analysis based on follow-up duration demonstrated that PBM was significantly more effective within the first two months of follow-up.

The limitations of this study must be acknowledged. First, although four types of HILT—Nd:YAG laser, CO₂ laser, Er:YAG laser, and Er,Cr:YSGG laser—were included, the limited number of relevant studies precluded subgroup analysis by laser type. Although HILT shows significant potential for the treatment of OLP, future RCTs should incorporate subgroup assessments based on laser type to more precisely delineate treatment effects. Second, the PBM group exhibited significant heterogeneity regarding pain, potentially stemming from variations in laser parameters, such as wavelength and treatment frequency. Subgroup analyses based on these parameters showed that the number of laser interventions may be an important factor affecting heterogeneity. However, PBM involves many adjustable parameters in clinical practice. Future studies should consider other factors to fully understand the sources of heterogeneity.Third, clinical scoring of OLP primarily utilized TSS and RAE. Despite the use of SMD for statistical analysis, the combination of TSS and RAE could introduce some heterogeneity. Finally, many of the studies had small sample sizes, and some data underwent transformation during analysis, which could have introduced inevitable bias. These results should therefore be interpreted with caution, and future research should aim to increase sample sizes to minimize the influence of bias.

Future research should concentrate on several key areas: First, standardization of application parameters such as power density, energy, frequency, and duration in HILT and PBM therapies is recommended to enhance comparability of results. Second, given the variety of methods to measure lesion severity, the development of an internationally recognized standard for OLP outcomes is necessary. Third, considering the potential additional costs of HILT and PBM compared to TCS, a cost-effectiveness analysis is advised to evaluate the economic viability of these therapies. Finally, as OLP is a chronic condition with a potential for malignancy, further research should focus on the psychological well-being of patients. Currently, only two studies [40, 41] have assessed depression and anxiety levels in OLP patients, which restricts our understanding of their psychological state.

## Conclusion

Current findings suggest that both HILT and PBM show promise in the short-term management of OLP, demonstrating improvements in relapse rates, cure rates, and clinical scores when compared to TCS.Regarding pain relief, HILT proved more effective than TCS, while PBM achieved comparable results to TCS.Notably, the studies reported no adverse reactions for PBM, whereas HILT was linked to mild local adverse effects, none of which were severe.Therefore, for OLP cases unresponsive to TCS, both HILT and PBM present potential alternative therapies.Given the limited availability and higher costs associated with RCTs for HILT, we recommend PBM therapy, with a total energy density not exceeding 120 J/cm^2^, as a preferable option for short-term OLP management.However, given that laser parameters vary across studies, caution is advised in the interpretation of these findings.Further large-scale RCTs are necessary to clarify the impact of specific laser settings and to assess the long-term efficacy and safety of HILT, PBM, and TCS.

## Supplementary Information

Below is the link to the electronic supplementary material.

ESM 1 (DOC 15.0 MB)

## Data Availability

Data is provided within the manuscript or supplementary information files.
