# Supplementary material for: Effect of high-intensity laser therapy and photobiomodulation therapy on oral lichen planus—a systematic review and meta-analysis
Source: Lasers Med Sci. 2025 Mar 20;40(1):151. doi: 10.1007/s10103-025-04398-8 (PMC11922975; doi:10.1007/s10103-025-04398-8)
Supplement: Supplementary file 1 — (DOC 15.0 MB) [file 10103_2025_4398_MOESM1_ESM.doc]

Effect of high-intensity laser therapy and photobiomodulation therapy on oral lichen planus - A systematic review and meta-analysis

This supplementary material has been provided by the authors to give readers additional information about their work.

**List of materials**

**Appendix 1** Differences between published and final protocol (P2)

**Appendix 2** Search strategy (P3-P4)

**Appendix 3** Supplementary tables and figures (P5 - P18)

**Appendix 1. Differences between published and final protocol**

- 1. **Title**：Refining Laser Interventions: Distinguishing Between High-Energy Lasers and Photobiomodulation (Low-Level Laser Therapy).

**Original Title:**Effect of laser therapy on oral lichen planus - A systematic review and meta-analysis

**Current Title:**Effect of high-intensity laser therapy and photobiomodulation therapy on oral lichen planus - A systematic review and meta-analysis

- 1. **Add two Chinese databases**: CNKI and SinoMed.
  2. **Update the search date**: Update the original search cutoff date from April 2024 to October 2024
  3. Outcomes added:

Clinical scores

Recurrence rates

Cure rates

Adverse events, such as clinical candidiasis, other toxic effects

- 1. **Types of study to be included**

We initially planned to collect randomized controlled trials (RCTs) on laser therapy for the treatment of oral lichen planus, as well as non-RCTs with a control group. However, the non-RCTs had a lower level of evidence. Therefore, we primarily included RCTs that utilized laser therapy for the treatment of oral lichen planus.

**Appendix 2 Search strategy**

**Pubmed**

("Lichen Planus, Oral"[Mesh] OR "oral lichen planus") AND ("Laser Therapy"[Mesh] OR "Lasers, Gas"[Mesh] OR "Lasers, Semiconductor"[Mesh] OR "Low-Level Light Therapy"[Mesh] OR "Lasers, Solid-State"[Mesh] OR "Lasers, Excimer"[Mesh]) OR

("Laser Surgery" OR "Laser Therapies" OR "laser treatment" OR "carbon dioxide laser" OR "CO2 Laser" OR "Diode Laser" OR "GaAlAs Laser" OR "GaAs Laser" OR "LLLT" OR "Low Level Laser Therapies" OR "Low Level Laser Therapy" OR "Photobiomodulation" OR "Photobiomodulation Therapy" OR "Er YAG Laser" OR "Excimer Laser" OR "Nd:YAG" OR "Er,Cr:YSGG")

**Cochrane search manager**

("Lichen Planus, Oral" OR "oral lichen planus") AND("Laser Therapy" OR "Lasers, Gas" OR "Lasers, Semiconductor" OR "Low-Level Light Therapy" OR "Lasers, Solid-State" OR "Lasers, Excimer") OR("Laser Surgery" OR "Laser Therapies" OR "laser treatment" OR "carbon dioxide laser" OR "CO2 Laser" OR "Diode Laser" OR "GaAlAs Laser" OR "GaAs Laser" OR "LLLT" OR "Low Level Laser Therapies" OR "Low Level Laser Therapy" OR "Photobiomodulation" OR "Photobiomodulation Therapy" OR "Er YAG Laser" OR "Excimer Laser" OR "Nd:YAG" OR "Er,Cr:YSGG")

**Web of science**

("oral lichen planus") AND("Laser Surgery" OR "Laser Therapies" OR "laser treatment" OR "carbon dioxide laser" OR "CO2 Laser" OR "Diode Laser" OR "GaAlAs Laser" OR "GaAs Laser" OR "LLLT" OR "Low Level Laser Therapies" OR "Low Level Laser Therapy" OR "Photobiomodulation" OR "Photobiomodulation Therapy" OR "Er YAG Laser" OR "Excimer Laser" OR "Nd:YAG" OR "Er,Cr:YSGG")

**Embase**

('Oral lichen planus'/exp OR 'oral lichen planus') AND ('Laser therapy'/exp OR 'laser therapy' OR 'laser treatment' OR 'laser surgery') OR('Carbon dioxide laser'/exp OR 'CO2 laser' OR 'carbon dioxide laser') OR('Diode laser'/exp OR 'diode laser') OR('Erbium YAG laser'/exp OR 'Er YAG laser' OR 'Erbium YAG laser') OR('Neodymium YAG laser'/exp OR 'Nd:YAG laser' OR 'neodymium YAG laser') OR('Low level laser therapy'/exp OR 'LLLT' OR 'low level laser therapy' OR 'low level laser therapies' OR 'Photobiomodulation' OR'photobiomodulation therapy') OR('Excimer laser'/exp OR 'excimer laser') OR('GaAlAs laser' OR 'GaAs laser' OR 'Er,Cr:YSGG laser')

**Appendix 3 Supplementary figure**

**Table S1. PRISMA 2020 checklist**

| **Section and Topic** | **Item #** | **Checklist item** | **Location where item is reported** |
| --- | --- | --- | --- |
| **TITLE** | | |  |
| Title | 1 | Identify the report as a systematic review. | Title (pg1) |
| **ABSTRACT** | | |  |
| Abstract | 2 | See the PRISMA 2020 for Abstracts checklist. | Abstract(pg1-2) |
| **INTRODUCTION** | | |  |
| Rationale | 3 | Describe the rationale for the review in the context of existing knowledge. | Introduction(pg2-3) |
| Objectives | 4 | Provide an explicit statement of the objective(s) or question(s) the review addresses. | Introduction(pg3) |
| **METHODS** | | |  |
| Eligibility criteria | 5 | Specify the inclusion and exclusion criteria for the review and how studies were grouped for the syntheses. | Methods(pg4) |
| Information sources | 6 | Specify all databases, registers, websites, organisations, reference lists and other sources searched or consulted to identify studies. Specify the date when each source was last searched or consulted. | Methods(pg4) |
| Search strategy | 7 | Present the full search strategies for all databases, registers and websites, including any filters and limits used. | Methods(pg4) |
| Selection process | 8 | Specify the methods used to decide whether a study met the inclusion criteria of the review, including how many reviewers screened each record and each report retrieved, whether they worked independently, and if applicable, details of automation tools used in the process. | Methods(pg5-6) |
| Data collection process | 9 | Specify the methods used to collect data from reports, including how many reviewers collected data from each report, whether they worked independently, any processes for obtaining or confirming data from study investigators, and if applicable, details of automation tools used in the process. | Methods(pg5-6) |
| Data items | 10a | List and define all outcomes for which data were sought. Specify whether all results that were compatible with each outcome domain in each study were sought (e.g. for all measures, time points, analyses), and if not, the methods used to decide which results to collect. | Methods(pg4-5) |
|  | 10b | List and define all other variables for which data were sought (e.g. participant and intervention characteristics, funding sources). Describe any assumptions made about any missing or unclear information. | Methods(pg4-5) |
| Study risk of bias assessment | 11 | Specify the methods used to assess risk of bias in the included studies, including details of the tool(s) used, how many reviewers assessed each study and whether they worked independently, and if applicable, details of automation tools used in the process. | Methods(pg5-6) |
| Effect measures | 12 | Specify for each outcome the effect measure(s) (e.g. risk ratio, mean difference) used in the synthesis or presentation of results. | Methods(pg6) |
| Synthesis methods | 13a | Describe the processes used to decide which studies were eligible for each synthesis (e.g. tabulating the study intervention characteristics and comparing against the planned groups for each synthesis (item #5)). | Methods(pg4-5) |
|  | 13b | Describe any methods required to prepare the data for presentation or synthesis, such as handling of missing summary statistics, or data conversions. | Methods(pg6) |
|  | 13c | Describe any methods used to tabulate or visually display results of individual studies and syntheses. | Methods(pg6) |
|  | 13d | Describe any methods used to synthesize results and provide a rationale for the choice(s). If meta-analysis was performed, describe the model(s), method(s) to identify the presence and extent of statistical heterogeneity, and software package(s) used. | Methods(pg6) |
|  | 13e | Describe any methods used to explore possible causes of heterogeneity among study results (e.g. subgroup analysis, meta-regression). | Methods(pg6) |
|  | 13f | Describe any sensitivity analyses conducted to assess robustness of the synthesized results. | Methods(pg6) |
| Reporting bias assessment | 14 | Describe any methods used to assess risk of bias due to missing results in a synthesis (arising from reporting biases). | Methods(pg6) |
| Certainty assessment | 15 | Describe any methods used to assess certainty (or confidence) in the body of evidence for an outcome. | Methods(pg5-6) |
| **RESULTS** | | |  |
| Study selection | 16a | Describe the results of the search and selection process, from the number of records identified in the search to the number of studies included in the review, ideally using a flow diagram. | Results(pg 7) |
|  | 16b | Cite studies that might appear to meet the inclusion criteria, but which were excluded, and explain why they were excluded. | Fig. 1, (pg 7) |
| Study characteristics | 17 | Cite each included study and present its characteristics. | Results(pg 8) |
| Risk of bias in studies | 18 | Present assessments of risk of bias for each included study. | Results(pg 8-9) |
| Results of individual studies | 19 | For all outcomes, present, for each study: (a) summary statistics for each group (where appropriate) and (b) an effect estimate and its precision (e.g. confidence/credible interval), ideally using structured tables or plots. | Results(pg 9-14) |
| Results of syntheses | 20a | For each synthesis, briefly summarise the characteristics and risk of bias among contributing studies. | Results(pg 9-14) |
|  | 20b | Present results of all statistical syntheses conducted. If meta-analysis was done, present for each the summary estimate and its precision (e.g. confidence/credible interval) and measures of statistical heterogeneity. If comparing groups, describe the direction of the effect. | Results(pg 9-14) |
|  | 20c | Present results of all investigations of possible causes of heterogeneity among study results. | Results(pg 14-15) |
|  | 20d | Present results of all sensitivity analyses conducted to assess the robustness of the synthesized results. | Results(pg 15) |
| Reporting biases | 21 | Present assessments of risk of bias due to missing results (arising from reporting biases) for each synthesis assessed. | Results(pg 14-15) |
| Certainty of evidence | 22 | Present assessments of certainty (or confidence) in the body of evidence for each outcome assessed. | Results(pg 8)，table 1. |
| **DISCUSSION** | | |  |
| Discussion | 23a | Provide a general interpretation of the results in the context of other evidence. | Disscussion(pg15-16) |
|  | 23b | Discuss any limitations of the evidence included in the review. | Disscussion(pg17-18) |
|  | 23c | Discuss any limitations of the review processes used. | Disscussion(pg17-18) |
|  | 23d | Discuss implications of the results for practice, policy, and future research. | Disscussion(pg17-18) |
| **OTHER INFORMATION** | | |  |
| Registration and protocol | 24a | Provide registration information for the review, including register name and registration number, or state that the review was not registered. | Pg3 |
|  | 24b | Indicate where the review protocol can be accessed, or state that a protocol was not prepared. | NA |
|  | 24c | Describe and explain any amendments to information provided at registration or in the protocol. | Pg3 |
| Support | 25 | Describe sources of financial or non-financial support for the review, and the role of the funders or sponsors in the review. | Pg1 |
| Competing interests | 26 | Declare any competing interests of review authors. | Pg19 |
| Availability of data, code and other materials | 27 | Report which of the following are publicly available and where they can be found: template data collection forms; data extracted from included studies; data used for all analyses; analytic code; any other materials used in the review. | Page 19 |

**Table S2 . Adverse events noted in the studies**

| Group | Study | Adverse events in the test group | Adverse events in the control group |
| --- | --- | --- | --- |
| HILT versus TCS | Wang et al.2022 | allotriogeusia; burning sensation; | allotriogeusia; burning sensation;sense of piercing pain;dry mouth;nausea;Candida infection; |
| HILT versus TCS | Zhong et al.2020 | adrenocortical insufficiency;proteolysis | fungal infection;adrenocortical insufficiency;proteolysis |
| PBM versus TCS | Dillenburg et al. 2014 | None | burning sensation;gastrointestinal discomfort |

PBM:Photobiomodulation; HILT:fhigh-level laser therapy;TCS:topical corticosteroids.

**Table S3. Analgesic Efficacy of PBM versus TCS in OLP Management**

| *s*ubgroup | Outcome | Number of  Studies Included | SMD (95%CI) | *P* value | heterogeneity |
| --- | --- | --- | --- | --- | --- |
| Symptomatic OLP | VAS | 5 | -0.73[-1.38, -0.09] | 0.026 | 75.3% |
| Erosive-Atrophic OLP | VAS | 4 | -0.01[-0.79, 0.78] | 0.985 | 81.3% |
| number of interventions  (≤10) | VAS | 8 | -0.35[-0.93,0.23] | 0.240 | 80.3% |
| number of interventions  (＞10) | VAS | 2 | -0.64[-1.01, -0.28] | 0.001 | 0% |
| laser wavelength  (＞800) | VAS | 6 | -0.40[-1.11, 0.30] | 0.260 | 84.6% |
| laser wavelength  (＜800) | VAS | 3 | -0.34[-0.99, 0.31] | 0.303 | 60.7% |
| PBM versus DMW | VAS | 2 | -0.26 [-0.75, 1.27] | 0.614 | 77.6% |
| PBM versus TA | VAS | 5 | -0.66 [-1.19, -0.13] | 0.015 | 70.2% |
| PBM versus 0.05% CP | VAS | 2 | -0.41 [-0.87, 0.04] | 0.511 | 80.3%, |

SMD = Standardized Mean Difference; CI = confidence interval; PBM =Photobiomodulation;TA :triamcinolone acetonide;DMW:Dexamethasone

CP:clobetasol propionate;VAS:Visual Analog Scale.

**
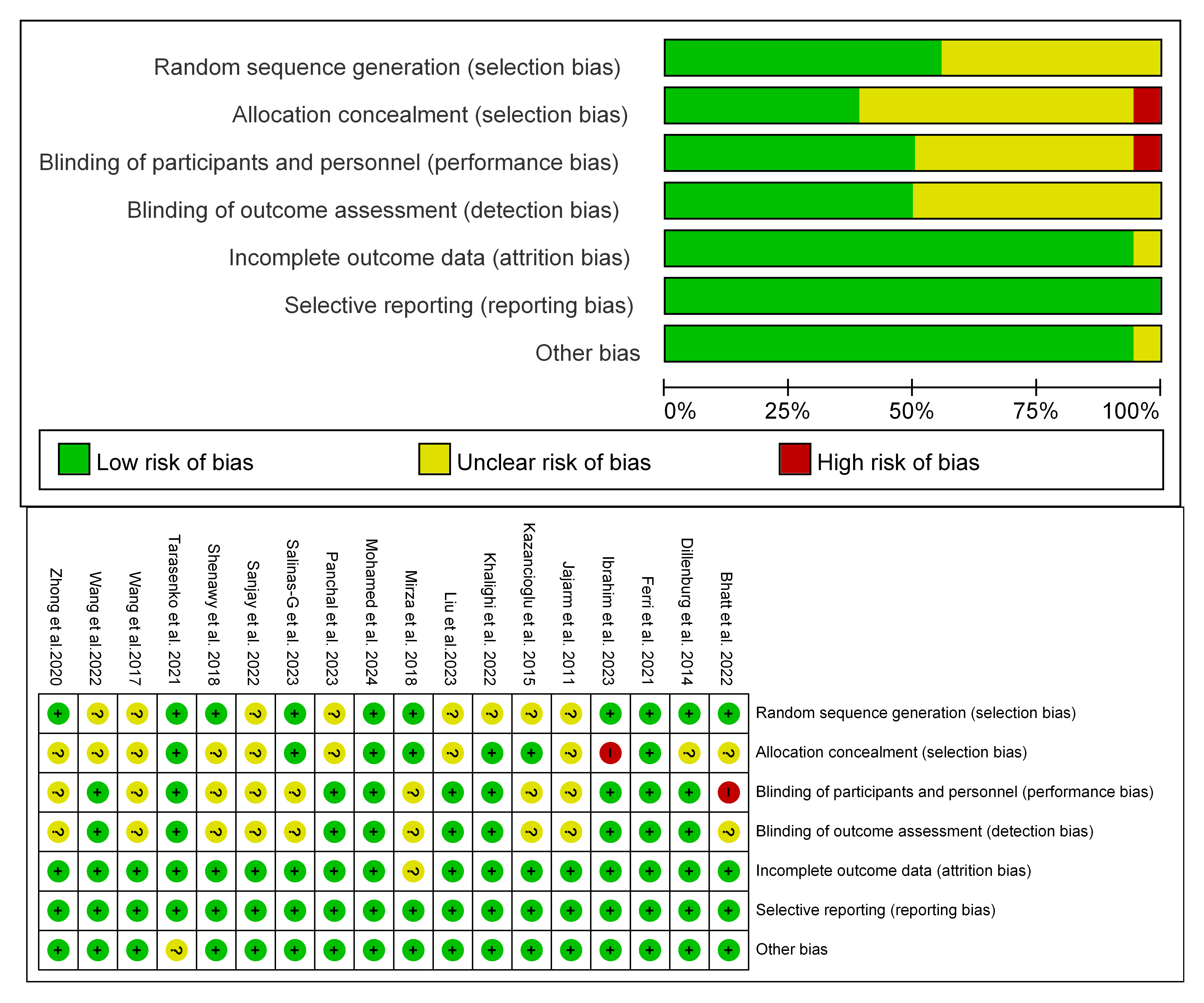
**

**Fig. S1 Risk of bias.A. Risk of bias graph; B. Risk of bias summary**

**
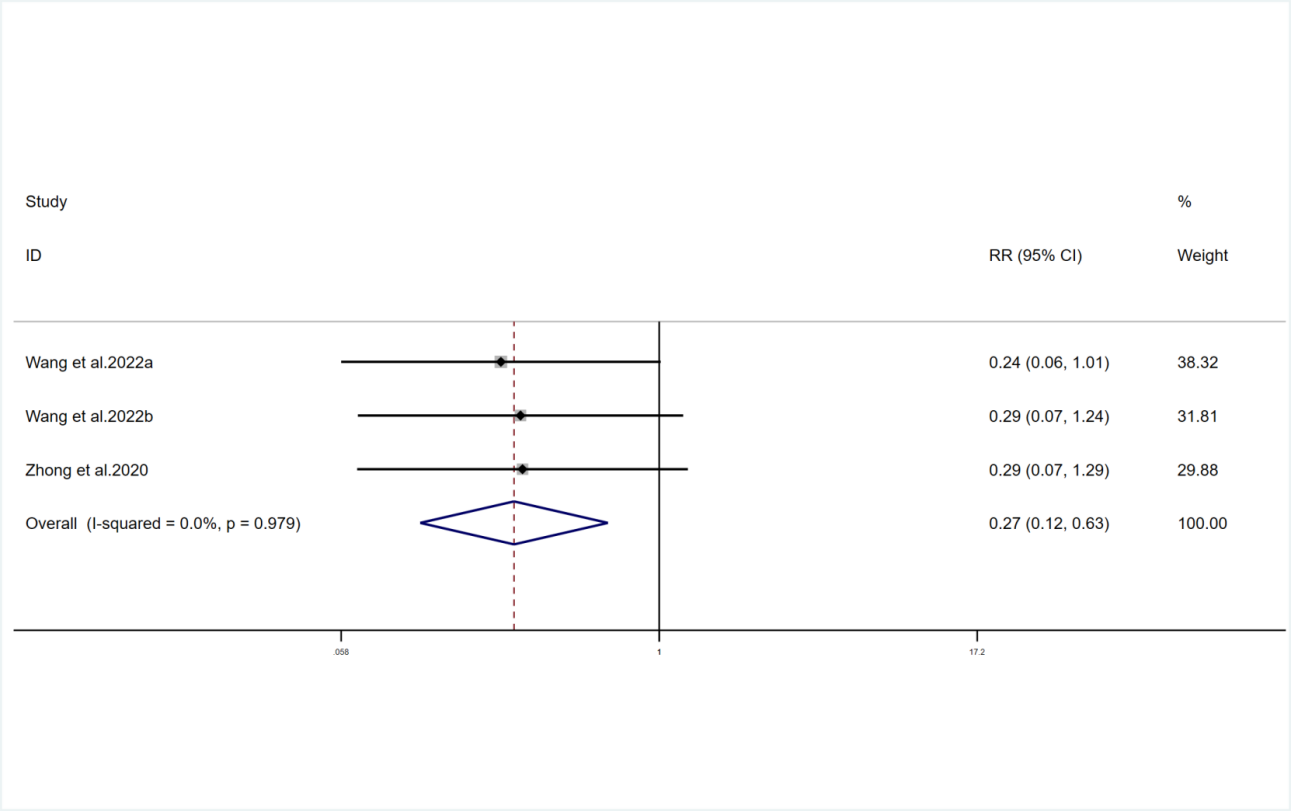
**

**Fig. S2 Forest plots of adverse events comparing HILT and TCS**

**
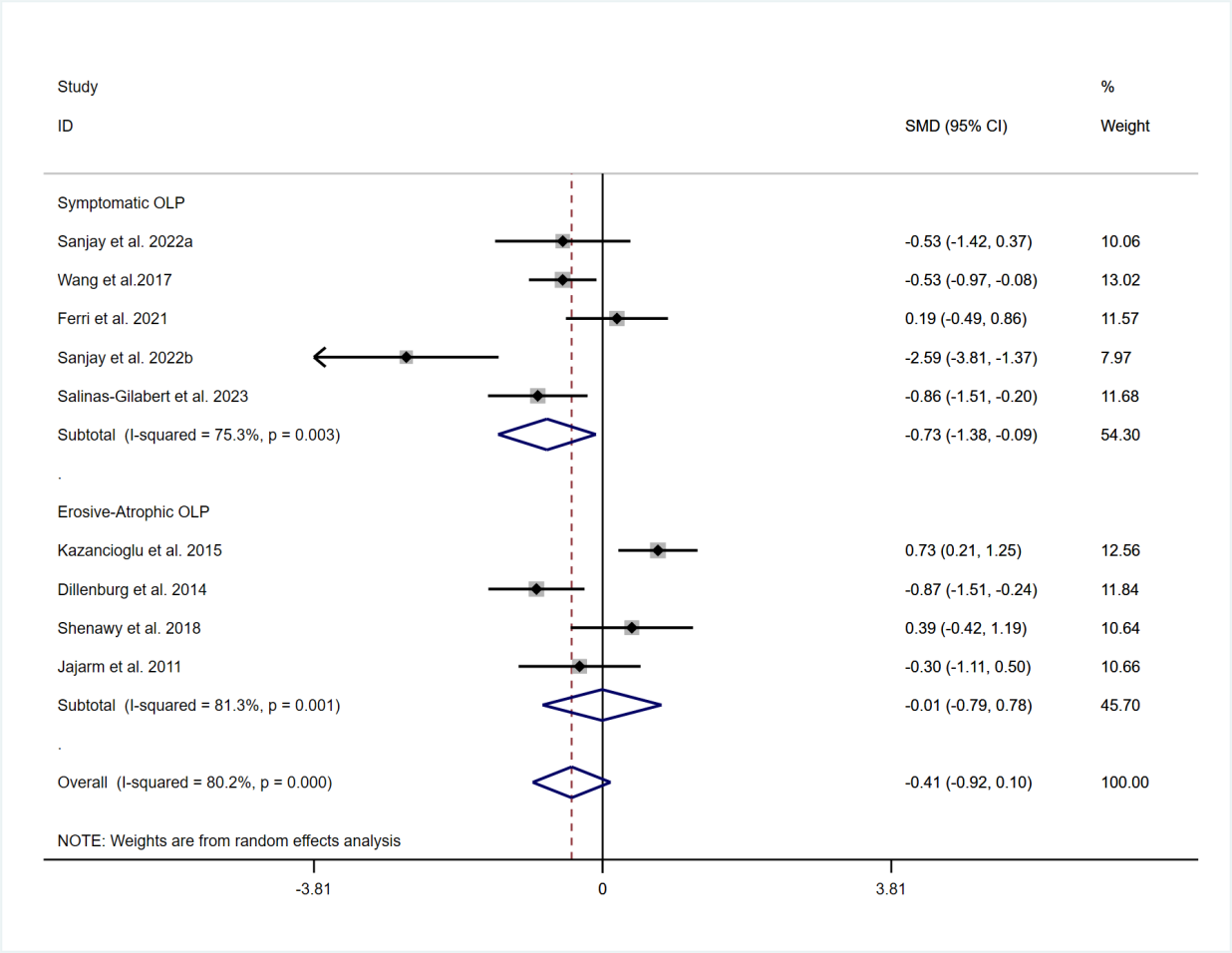
**

**Fig. S3 Forest plot of VAS score by OLP type**


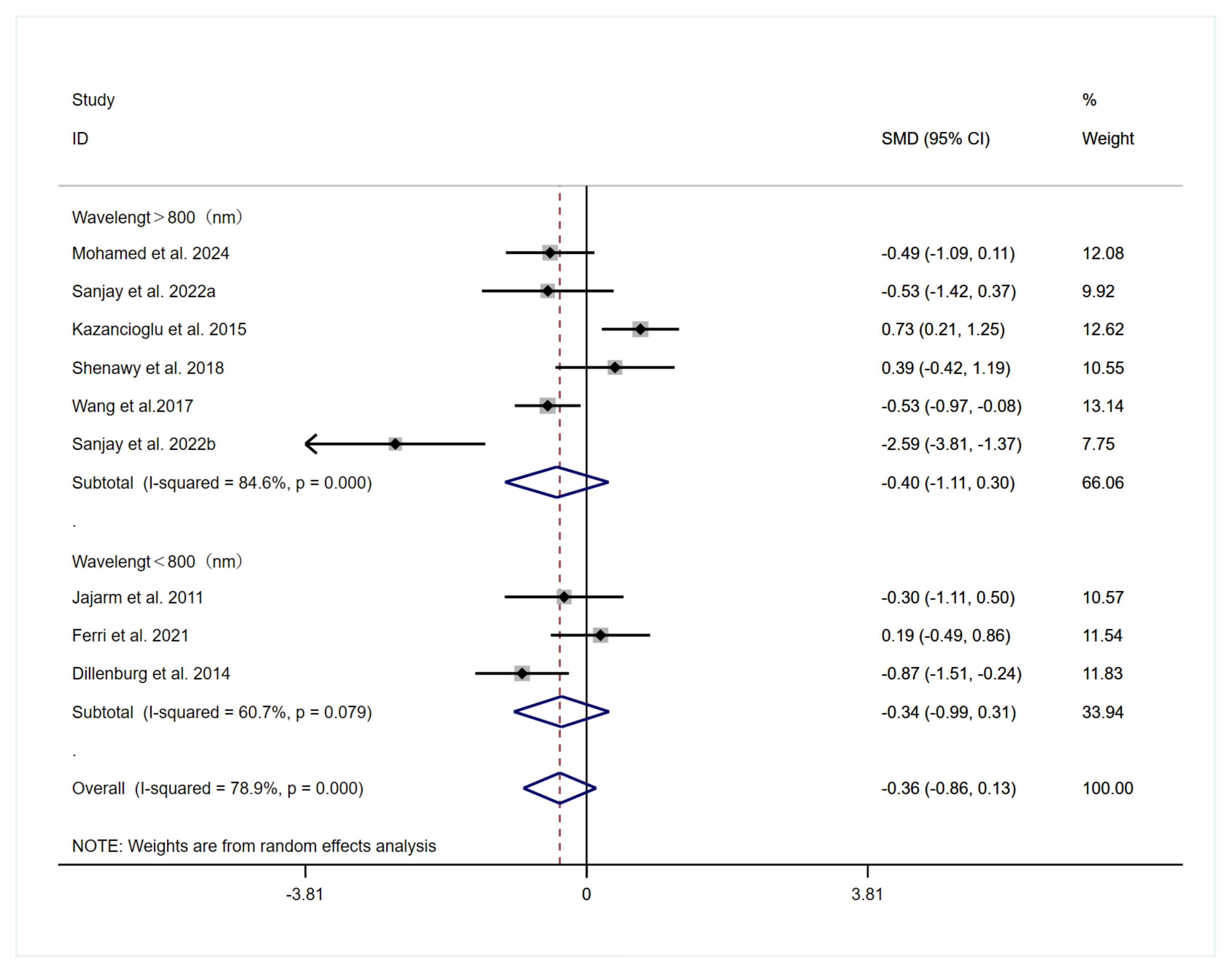


**Fig. S4 Forest plot of VAS score by laser wavelength classification**


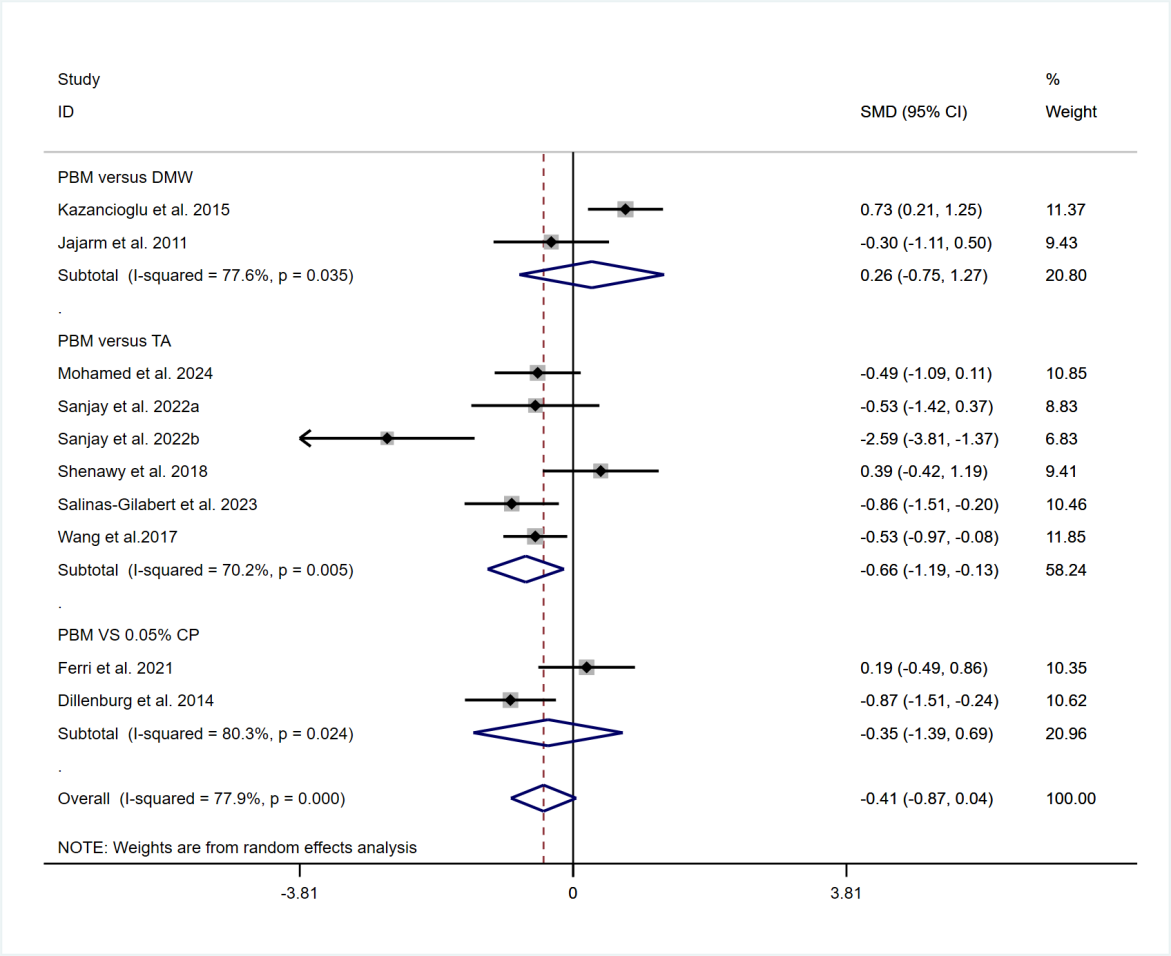


**Fig. S5 Forest plot of VAS score by types of topical corticosteroids**


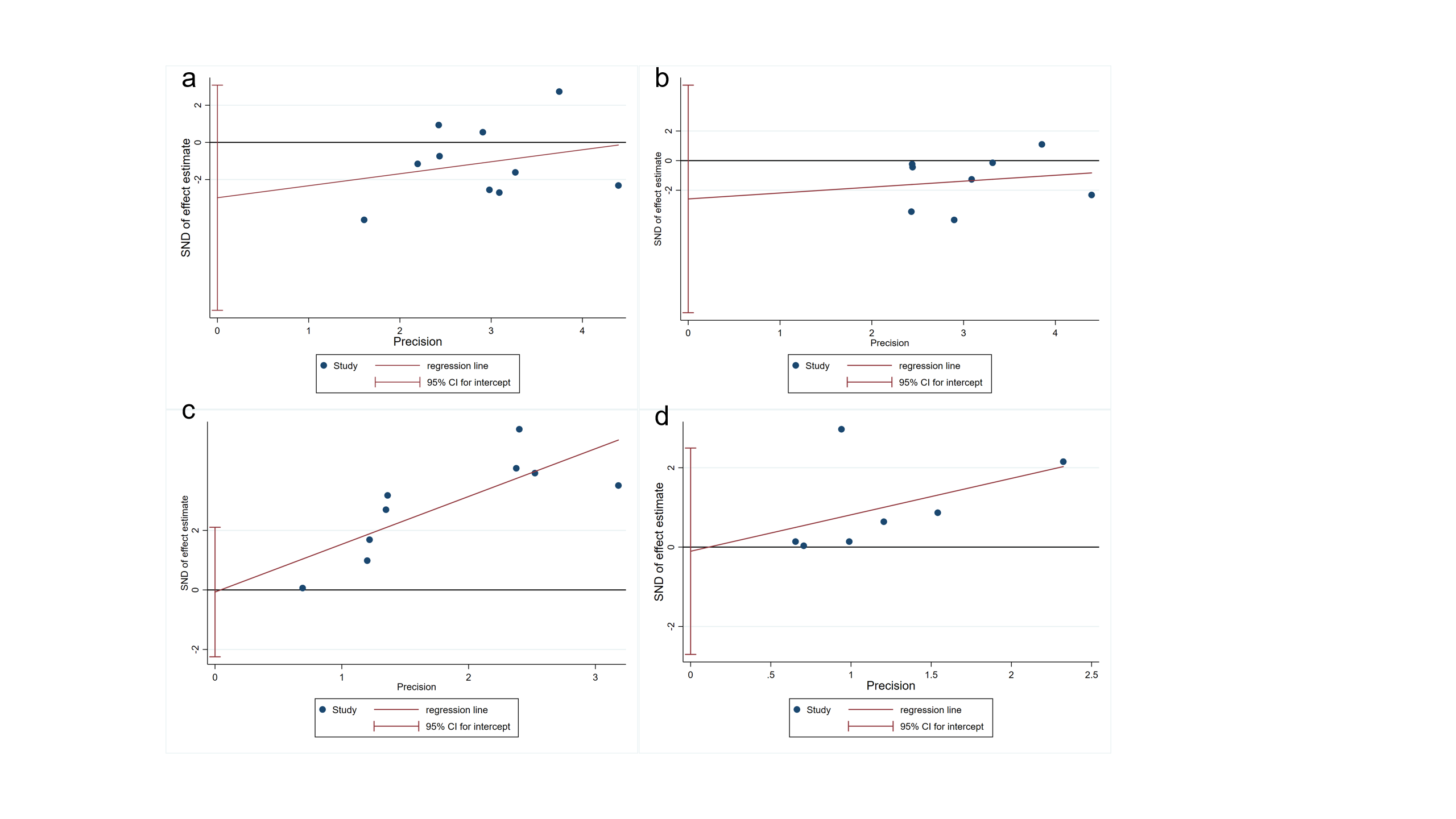
**Fig .S6 Egger’s test of (a) VAS score, (b) clinical score, (c) cure rate, and (d) recurrence rate comparing PBM and TCS**


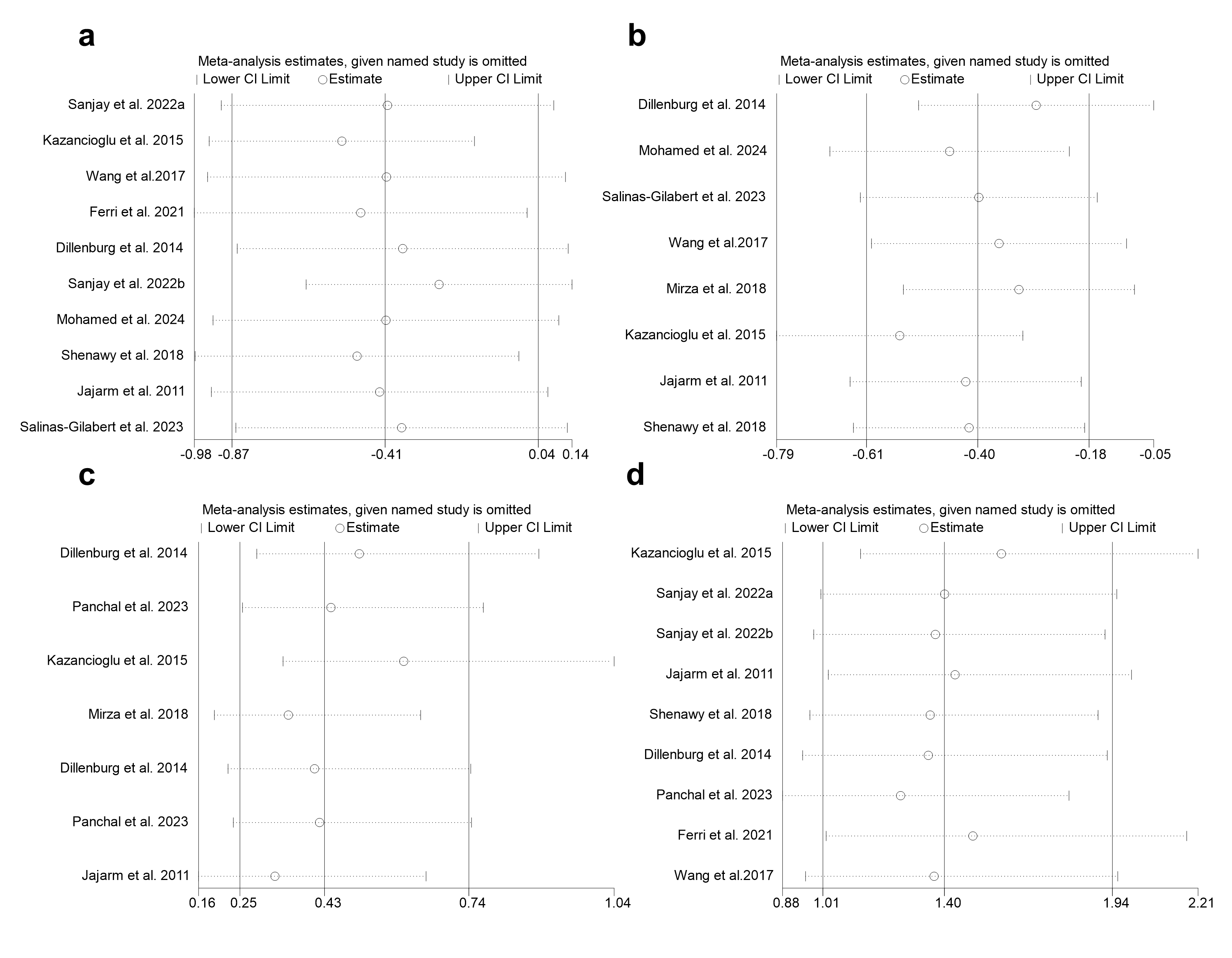
**Fig. S7 Sensitivity analysis of (a) VAS score, (b) clinical score, (c) cure rate, and (d) recurrence rate comparing PBM and TCS**


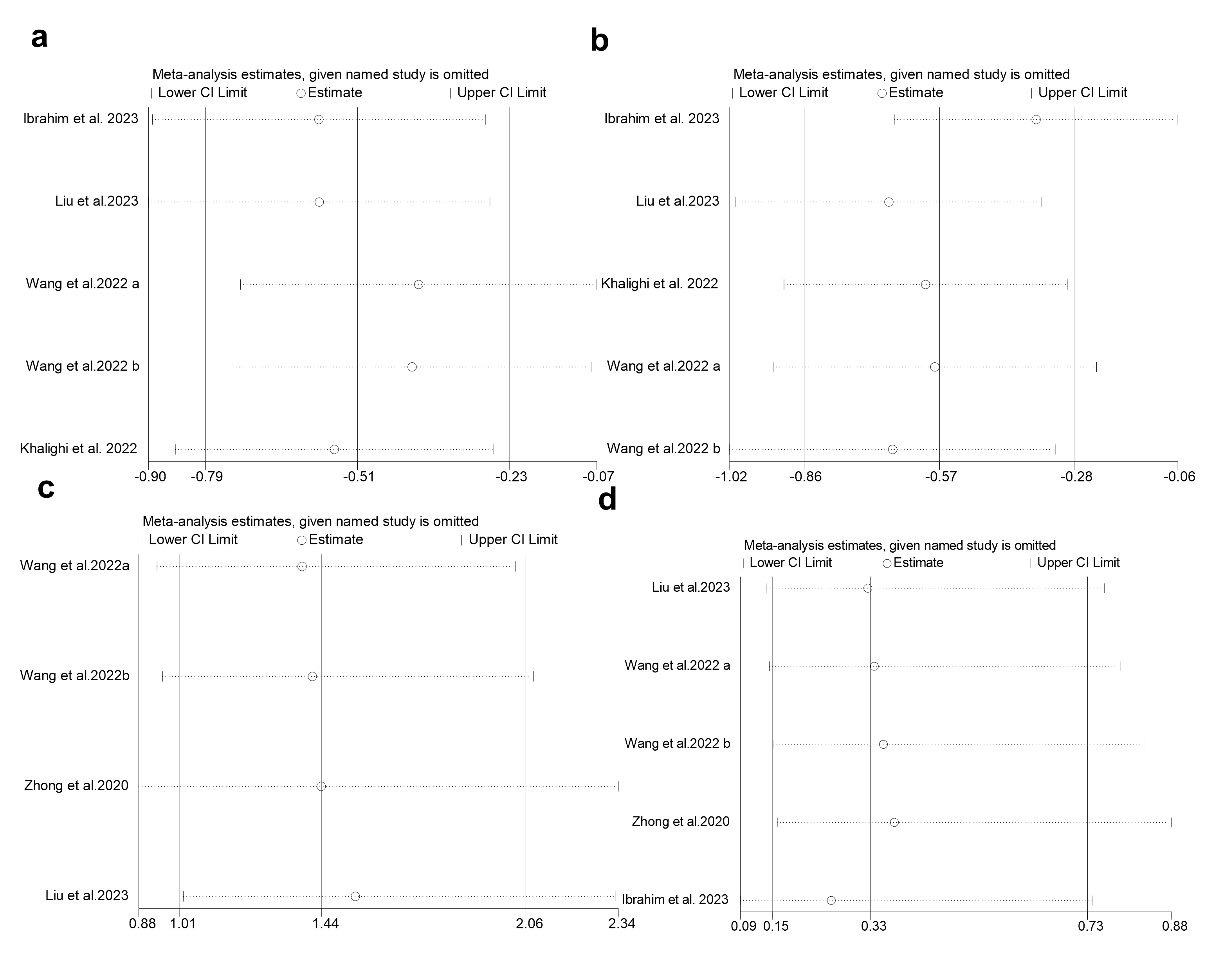
**Fig. S8 Sensitivity analysis of (a) VAS score, (b) clinical score, (c) cure rate, and (d) recurrence rate comparing HILT and TCS**
